# Comparing the self-perceived quality of life of multimorbid patients and the general population using the EQ-5D-3L

**DOI:** 10.1371/journal.pone.0188499

**Published:** 2017-12-19

**Authors:** Alexandra A. N’Goran, Anouk Déruaz-Luyet, Dagmar M. Haller, Andreas Zeller, Thomas Rosemann, Sven Streit, Lilli Herzig

**Affiliations:** 1 Institute of Family Medicine, University of Lausanne, Lausanne, Switzerland; 2 Primary Care Unit, Faculty of Medicine, University of Geneva, Geneva, Switzerland; 3 Centre for Primary Health Care, University of Basel, Basel, Switzerland; 4 Institute of Primary Care, University of Zurich, Zurich, Switzerland; 5 Institute of Primary Health Care (BIHAM), University of Bern, Bern, Switzerland; La Trobe University, AUSTRALIA

## Abstract

**Objectives:**

To assess and compare the self-perceived Health Related Quality of Life (HRQoL) of multimorbid patients and the general population using health utilities (HU) and visual analogue scale (VAS) methods.

**Methods:**

We analyzed data (n = 888) from a national, cross-sectional Swiss study of multimorbid patients recruited in primary care settings. Self-perceived HRQoL was assessed using the EQ-5D-3L instrument, composed of 1) a questionnaire on the five dimensions of mobility, self-care, usual activities, pain/discomfort, and anxiety/depression (EQ-5D dimensions), and 2) a 0–100 (0 = worst- and 100 = best-imaginable health status) VAS. We described the EQ-5D dimensions and VAS and computed HU using a standard pan-European value set. HU and VAS are the two components of the overall HRQoL assessment. We examined the proportions of multimorbid patients reporting problems (moderate/severe) in each EQ-5D dimension, corresponding proportions without problems, and mean HU and VAS values across patient characteristics. To test differences between subgroups, we used chi-square tests for dichotomous outcomes and *T*-tests (ANOVA if more than two groups) for continuous outcomes. Finally, we compared observed and predicted HU and VAS values.

**Results:**

All 888 participants answered every EQ-5D item. Mean (SD) HU and VAS values were 0.70 (0.18) and 63.2 (19.2), respectively. HU and VAS were considerably and significantly lower in multimorbid patients than in the general population and were also lower in multimorbid patients below 60 years old and in women. Differences between observed and predicted means (SD) were -0.07 (0.18) for HU and -11.8 (20.3) for VAS.

**Conclusions:**

Self-perceived HRQoL is considerably and significantly affected by multimorbidity. More attention should be given to developing interventions that improve the HRQoL of multimorbid patients, particularly women and those aged below 60 years old.

## Introduction

Multimorbidity, commonly defined as the co-occurrence of two or more chronic conditions in one individual [1–3], is increasing due to improved living conditions, better medical care, and an aging population [4, 5]. Multimorbidity is associated with increased rates of mortality and disability, reduced levels of function, increased polypharmacy, poor health-related quality of life (HRQoL), and a greater utilization of healthcare resources (costs, number of physician visits, length of hospital stay) [6]. Indeed, multimorbidity is recognized as a significant healthcare system cost and a major public health issue deserving more research [7]. As multimorbidity becomes more prevalent worldwide, it is becoming a more relevant, clinically important topic. In Switzerland, multimorbid patients are estimated to make up 20%–30% of the general population [8].

The cumulative effects of multiple chronic conditions on an individual are complex; specific combinations of conditions may have greater effects on functional status, quality of life (QoL), and mortality than others [9, 10]. Several studies have shown that multimorbidity is significantly associated with poor HRQoL in adult patients [11–13]. However, little is known about multimorbid patients’ self-perceived HRQoL in comparison with the general population and in different multimorbid patient age groups.

We aimed to assess multimorbid patients’ self-perceived QoL and compare the results with findings from a general population by using two components of the EQ-5D-3L instrument: health utilities (HU) and visual analogue scale (VAS) scores. This will provide information to guide future clinical and policy decision-making for the better care of multimorbid patients in primary care in Switzerland. The EQ-5D-3L is a widely used generic instrument to measure and evaluate health status; it provides a simple descriptive profile and a single index for health status that can be used in the clinical and economic evaluation of healthcare, as well as in population health surveys [14].

The hypothesis was that multimorbid patients might suffer from more limitations to the physical and functional activities that have an impact on their HU and VAS than to the general population. A further hypothesis was that the QoL of older multimorbid patients would be worse than that of younger multimorbid patients. Although several studies have assessed the QoL of multimorbid patients, to the best of our knowledge, none have ever assessed their HU and VAS using the EQ-5D-3L instrument and compared them to the general population. The present study aimed to fill this gap.

## Materials and methods

### Participants and procedures

We analyzed data (n = 888) from a national cross-sectional survey conducted in collaboration with Switzerland’s five Institutes of Family Medicine, between January and September 2015. That study was designed to assess multimorbidity in patients in primary care settings. The detailed study protocol and its initial results have been published elsewhere [15, 16].

Briefly, a convenience sample of 100 general practitioners (GPs) randomly enrolled multimorbid participants attending their practices during the study period. Each GP was provided with a randomization calendar specifying which patients to enroll during each half-day of the recruitment weeks. Eligible patients were aged 18 years old and over and had at least three chronic conditions on a list of 75 (“S Tabe 1”) [17]. The GPs informed each eligible patient about the study and its procedures and asked for their written consent. The study protocol (Protocol No 314/15) was approved by the Human Research Ethics Committee of the Canton Vaud.

### Measures

#### The EQ-5D-3L

Self-perceived QoL was assessed using the EQ-5D-3L instrument [14, 18], a widely used, two-part generic instrument for measuring and evaluating HRQoL [14, 19–21].

- The first part is a questionnaire of EQ-5D items (relating to the five dimensions of mobility, self-care, usual activities, pain/discomfort, and anxiety/depression) that is frequently used to measure HU in medical and public health research [20, 21]. Participants report their perceived health status by rating each dimension, using a three-level ordinal scale, as “no problem” (scored 1), a “moderate problem” (scored 2), or a “severe problem” (scored 3). This results in a total of 243 (i.e., 3^5^) different possible combinations of health states for mobility, self-care, usual activities, pain/discomfort, and anxiety/depression. A result of `11121`means that four EQ-5D dimensions were rated “no problem”, but that pain/discomfort was rated as a “moderate problem”. The five dimensions can also be combined into a single measure by using a unified scoring algorithm derived from the time trade-off data from several European studies [14, 20–22]. HU are self-reported overall measures of HRQoL; they are measured on a scale between 0 (representing a state of death) and 1 (representing perfect health) [21, 23]. Perneger et al. provided reference values for the general population (in French-speaking Switzerland) which can be used to assess the utility loss of specific health states.

- The second part of the EQ-5D-3L instrument is a visual analog scale (VAS), presented like a “thermometer” ranging from 0 to 100, and used to assess self-perceived global levels of health, where 0 represents the worst imaginable state of health and 100 represents the best imaginable state [20, 21].

#### Covariates

Demographic covariates included age, sex (female/male), marital status (single, married, divorced, or widow/er), education (compulsory education, upper secondary level, tertiary level), language (French/German), number of chronic conditions (3–4, 5–6, or ≥ 7), number of prescribed medicines (0–4, 5–6, 7–9, or ≥ 10), number of medical visits in the last month (1–2, 3–4, or ≥ 5), hospitalization in the last year (yes/no), and the severity index (low = low severity of organ dysfunction, or high = high severity of organ dysfunction). The severity index is derived from the cumulative illness rating scale (CIRS) [24]. The CIRS is a validated questionnaire grading the scale of impairment in each organ system, from 0–4 in 14 categories. GPs were asked to rate each participating patient across the different categories of the CIRS. The severity index (SI) [25–27] was obtained by dividing the total score by the number of categories with morbidities.

### Statistical analyses

We performed four main analyses. First, we described the EQ-5D items and the VAS in multimorbid patients. Second, we computed the HU for each multimorbid patient using a standard pan-European value set [14]. For each of the five EQ-5D items, we dichotomized the population into multimorbid patients with a problem (moderate/severe) and multimorbid patients without a problem, examined the proportions for each health domain, and calculated mean HU and mean VAS values across patient characteristics (i.e., age, sex, marital status, language, education, medical visits in the last month, hospitalizations in the last year, number of chronic conditions, number of medicines prescribed, and severity index derived from the CIRS) [24]. To test differences between subgroups in univariate analyses, we used chi-square tests for dichotomous outcomes and Welch *T*-tests (ANOVA if more than two groups) for continuous outcomes. Third, we examined the frequencies of the most common health states among the 243 possible combinations for each of the EQ-5D domains [14].

Finally, to compare the estimated HU and VAS values in our sample with those of the general population, we used the previously published reference values generated from 1,952 randomly selected adults in the French-speaking part of Switzerland [21].

All analyses were conducted using Stata software, version 14 (StataCorp LP, College Station, TX, USA).

## Results

The mean age (SD) of the 888 participants was 72.9 (12.0) years (range 28–98); 51.8% were women; 49.2% were married; 38.3% were French-speaking; 23.3% had more than seven chronic conditions; and 83.1% had a low severity index (i.e., low severity of organ dysfunction).

All 888 participants answered all five EQ-5D items, and the most frequently reported response in the domains of mobility (56%), self-care (88.4%), usual activities (61.2%), and anxiety/depression (58.1%) was “no problem”. For pain/discomfort, most participants reported a moderate problem (66.5%).

The mean (SD) HU value was 0.70 (0.18). The mean (SD) VAS value was 63.2 (19.2), with a minimum of 0 and a maximum of 100. Mean VAS of each three-point scale for mobility, self-care, usual activities, pain/discomfort, and anxiety/depression dimensions are presented in Table 1.

**Table 1:** Distributions of the EQ-5D-3L (EQ-5D items and visual analog scale) among multimorbid patients. N = 888.

EQ-5D	N (%)	VAS (SD)
**Mobility**		
No problem	497 (56.0)	68.7 (18.0)
Moderate problem	386 (43.5)	56.5 (18.4)
Severe problem	5 (0.6)	34.0 (18.2)
**Self-care**		
No problem	785 (88.4)	65.1 (18.1)
Moderate problem	92 (10.4)	49.6 (21.1)
Severe problem	11 (1.2)	41.8 (22.6)
**Usual activities**		
No problem	543 (61.2)	69.3 (17.1)
Moderate problem	328 (36.9)	54.7 (17.4)
Severe problem	17 (1.9)	31.8 (23.9)
**Pain/discomfort**		
No problem	211 (23.8)	74.1 (16.5)
Moderate problem	591 (66.5)	61.7 (17.4)
Severe problem	86 (9.7)	46.5 (22.1)
**Anxiety/depression**		
No problem	516 (58.1)	68.2 (17.7)
Moderate problem	329 (37.0)	57.8 (18.1)
Severe problem	43 (4.9)	44.2 (22.9)

VAS: visual analog scale

Only 68 of the 243 different possible health states were represented in our sample, with 30 health states selected relatively more often (i.e., with n ≥ 5) and 38 selected at “other” frequencies, i.e., all the health states with n < 5 participants (of these, 25 health states were only selected by one participant each). The most represented health state in our sample was “11121” (n = 143), i.e. “no problem” with mobility, self-care, usual activities, and anxiety/depression, and “moderate problems” with pain/discomfort. The best possible health state (i.e., “11111” and a “no problem” rating for all five EQ-5D items) was reported by 121 participants (13.6%); their mean HU value was 0.98, and their mean (SD) VAS was 78 (14.2). The health state represented by “22232” (i.e., a “moderate problem” with four EQ-5D items, but “severe problems” with pain/discomfort) was selected by seven participants; their health state had the lowest mean HU value (0.26) and mean VAS (40.0). The most frequently selected EQ-5D health states and the distributions of their corresponding HU and VAS values are presented in Table 2.

**Table 2:** Most frequently reported EQ-5D health states and distributions of corresponding mean HU and mean VAS values among a sample of multimorbid patients. N = 888.

Health state*	N (%)	Health utility	Mean VAS (SD)
11121	143 (16.1)	0.78	70.7 (16.4)
11111	121 (13.6)	0.98	78.0 (14.2)
21121	69 (7.8)	0.72	64.6 (15.5)
11122	66 (7.4)	0.70	66.1 (15.8)
21222	66 (7.4)	0.62	55.6 (13.4)
21221	48 (5.4)	0.70	58.5 (14.9)
21122	39 (4.4)	0.65	59.3 (15.6)
11222	36 (4.0)	0.68	58.4 (17.5)
11112	35 (3.9)	0.79	71.7 (17.3)
11221	31 (3.5)	0.76	63.2 (14.0)
22222	21 (2.4)	0.52	47.4 (13.7)
21111	19 (2.1)	0.81	68.7 (19.0)
21232	15 (1.7)	0.36	40.7 (12.7)
11123	12 (1.3)	0.47	55.0 (19.3)
22221	11(1.2)	0.60	58.2 (8.7)
21131	10 (1.1)	0.46	59.5 (19.8)
21231	10 (1.1)	0.44	56.0 (22.8)
21223	8 (0.9)	0.39	51.9 (16.5)
22232	7 (0.8)	0.26	40.0 (23.3)
21211	7 (0.8)	0.78	72.1 (14.7)
11232	6 (0.7)	0.42	49.2 (11.1)
11212	6 (0.7)	0.76	64.2 (17.4)
11131	6 (0.7)	0.52	67.5 (17.2)
11211	6 (0.7)	0.84	60.8 (17.0)
12221	6 (0.7)	0.66	62.7 (16.3)
21112	5 (0.6)	0.73	67.0 (12.0)
22121	5 (0.6)	0.62	74.0 (11.4)
12222	5 (0.6)	0.58	54.0 (8.9)
22231	5 (0.6)	0.34	57.0 (17.9)
21233	5 (0.6)	0.33	43.0 (31.1)
Other^ǂ^	59 (6.6)	**—**	**—**

*The health-scale column’s numbers represent scores for mobility, self-care, usual activities, pain/discomfort, and anxiety/depression (1 = no problem, 2 = moderate problem, 3 = severe problem). *For example*, *“11121” means that all the EQ-5D items were rated “no problem”*, *except for pain/discomfort*, *which was rated “moderate problem”*.

^ǂ^ Other regroups includes all the less frequently reported health states (n < 5)

VAS: visual analog scale

In the overall sample of multimorbid patients, the proportions with a moderate or severe problem were 76.24% for pain/discomfort, 44% for mobility, 41.9% for anxiety/depression, 38.8% for daily activities, and 11.6% for self-care. Women reported significantly more problems than men in all dimensions except self-care (*p* = 0.73). Patients aged > 80 years old reported more problems with mobility (*p* < 0.0001); patients aged < 60 years old reported more problems with daily activities (*p* = 0.02) and anxiety/depression (*p* < 0.0001). Patients hospitalized in the last year, with a high number of chronic conditions, and with a high severity index reported more problems in all five dimensions.

With regards to means for HU and VAS, significant differences were found according to sex, i.e., women had lower mean HU and VAS scores than men (*p* < 0.0001), and patients < 60 years old had lower mean HU and VAS than older patients (*p* < 0.0001). The comparisons between different subgroups of multimorbid patients are presented in Table 3.

**Table 3:** Univariate subgroup comparisons in the sample of multimorbid patients. N = 888.

	N (%)	No problem N (%)	Problem with mobility N (%)	Problem with self-care N (%)	Problem with daily activities N (%)	Problem with pain or discomfort N (%)	Problem with anxiety or depression N (%)	Mean health utility (SD)	Mean VAS (SD)
**Total**	888 (100)	121 (13.6)	391 (44.0)	103 (11.6)	345 (38.9)	677 (76.2)	372 (41.9)	0.70 (0.18)	63.2 (19.3)
**Sex**		*p* < 0.0001	*p* = 0.0125	*p* = 0.7302	*p* < 0.0001	*p* < 0.0001	*p* < 0.0001	*p* < 0.0001	*p* < 0.0001
Men	428 (48.2)	86 (20.1)	170 (39.7)	48 (11.2)	133 (31.1)	301 (70.3)	134 (31.3)	0.73 (0.18)	66.0 (18.8)
Women	460 (51.8)	35 (7.6)	221 (48.0)	55 (12.0)	212 (46.1)	376 (81.7)	238 (51.7)	0.67 (0.17)	60.5 (19.3)
**Age group**		*p* = 0.0814	*p* = 0.0002	*p* = 0.3479	*p* = 0.0194	*p* = 0.7456	*p* < 0.0001	*p* < 0.0001	*p* < 0.0001
< 60	128 (14.4)	13 (10.2)	47 (36.7)	17 (13.3)	63 (49.2)	100 (78.1)	93 (72.7)	0.63 (0.21)	55.7 (22.0)
60–79	482 (54.3)	77 (16.0)	194 (40.2)	49 (10.2)	172 (35.7)	369 (76.6)	186 (38.6)	0.71 (0.18)	65.4 (18.7)
≥ 80	278 (31.3)	31 (11.2)	150 (54.0)	37 (13.3)	110 (39.6)	208 (74.8)	93 (33.5)	0.71 (0.15)	62.9 (17.9)
**Marital status**		*p* = 0.0214	*p* = 0.0001	*p* = 0.3405	*p* = 0.0038	*p* = 0.4708	*p* = 0.0009	*p* = 0.0021	*p* = 0.0001
Single	85 (9.6)	8 (9.4)	31 (36.5)	9 (10.6)	30 (35.3)	65 (76.5)	47 (55.3)	0.69 (0.17)	62.6 (20.6)
Married	437 (49.2)	71 (16.2)	172 (39.4)	43 (9.8)	149 (34.1)	328 (75.1)	159 (36.4)	0.72 (0.17)	65.6 (18.7)
Divorced	150 (16.9)	24 (16.0)	65 (43.3)	21 (14.0)	75 (50.0)	111 (74.0)	76 (50.7)	0.66 (0.21)	57.4 (19.5)
Widow/er	216 (24.3)	18 (8.3)	123 (56.9)	30 (13.9)	91 (42.1)	173 (80.1)	90 (41.7)	0.69 (0.16)	62.6 (18.9)
Education*		*p* = 0.5804	*p* = 0.5926	*p* = 0.8371	*p* = 0.5093	*p* = 0.4651	*p* = 0.1999	*p* = 0.3000	*p* = 0.3562
Compulsory education	195 (22.0)	23 (11.8)	92 (47.2)	25 (12.8)	70 (35.9)	155 (79.5)	90 (46.2)	0.68 (0.18)	61.9 (19.0)
Upper secondary level	337 (38.0)	45 (13.4)	145 (43.0)	38 (11.3)	138 (40.9)	255 (75.7)	145 (43.0)	0.70 (0.17)	62.8 (18.7)
Tertiary level	355 (40.0)	53 (14.9)	153 (43.1)	40 (11.3)	137 (38.6)	266 (74.9)	137 (38.6)	0.71 (0.18)	64.3 (19.9)
**Medical visits in the last month**		*p* = 0.3543	*p* = 0.1171	*p* = 0.0465	*p* = 0.0215	*p* = 0.1960	*p* = 0.6719	*p* = 0.7038	*p* = 0.5885
1–2 visits	692 (77.9)	100 (14.5)	292 (42.2)	77 (11.1)	255 (36.8)	525 (75.9)	292 (42.2)	0.70 (0.18)	63.5 (19.6)
3–4 visits	141 (15.9)	14 (9.9)	71 (50.4)	14 (9.9)	60 (42.6)	114 (80.9)	55 (39.0)	0.69 (0.17)	62.9 (19.3)
≥ 5 visits	55 (6.2)	7 (12.7)	28 (50.9)	12 (21.8)	30 (54.5)	38 (69.1)	25 (45.5)	0.69 (0.19)	60.7 (14.4)
Hospitalization**		*p* = 0.3375	*p* = 0.0054	*p* = 0.0037	*p* = 0.0031	*p* = 0.4352	*p* = 0.6029	*p* = 0.0095	*p* = 0.0083
No	593 (67.0)	85 (14.3)	242 (40.8)	56 (9.4)	211 (35.6)	449 (75.7)	245 (41.3)	0.71 (0.17)	64.4 (18.9)
Yes	292 (33.0)	35 (12.0)	148 (50.7)	47 (16.1)	134 (45.9)	228 (78.1)	126 (43.2)	0.68 (0.19)	60.7 (19.7)
**Number of CC**		*p* = 0.0003	*p* < 0.0001	*p* < 0.0001	*p* = 0.0002	*p* = 0.0022	*p* = 0.8274	*p* < 0.0001	*p* = 0.0701
3–4	343 (38.6)	63 (18.4)	111 (32.4)	26 (7.6)	110 (32.1)	245 (71.4)	140 (40.8)	0.73 (0.17)	64.7 (18.5)
5–6	338 (38.1)	45 (13.3)	162 (47.9)	35 (10.4)	132 (39.1)	257 (76.0)	142 (42.0)	0.70 (0.17)	63.1 (19.8)
≥7	207 (23.3)	13 (6.3)	118 (57.0)	42 (20.3)	103 (49.8)	175 (84.5)	90 (43.5)	0.65 (0.18)	60.8 (19.3)
**Number of medicines**		*p* < 0.0001	*p* < 0.0001	*p* = 0.0015	*p* < 0.0001	*p* < 0.0001	*p* = 0.1096	*p* < 0.0001	*p* < 0.0001
0–4	156 (17.6)	31 (19.9)	46 (29.5)	10 (6.4)	44 (28.2)	104 (66.7)	60 (38.5)	0.75 (0.16)	68.9 (17.9)
5–6	212 (23.9)	39 (18.4)	82 (38.7)	17 (8.0)	67 (31.6)	148 (69.8)	82 (38.7)	0.74 (0.16)	66.6 (19.2)
7–9	276 (31.1)	40 (14.5)	119 (43.1)	33 (12.0)	110 (39.9)	213 (77.2)	112 (40.6)	0.70 (0.18)	62.2 (17.9)
10–21	244 (27.5)	11 (4.5)	144 (59.0)	43 (17.6)	124 (50.8)	212 (86.9)	118 (48.4)	0.63 (0.18)	57.6 (20.0)
**Severity index**		*p* = 0.7071	*p* < 0.0001	*p* = 0.0648	*p* = 0.0068	*p* = 0.9399	*p* = 0.1935	*p* = 0.0060	*p* = 0.0005
Low	738 (83.1)	102 (13.8)	302 (40.9)	79 (10.7)	272 (36.9)	563 (76.3)	302 (40.9)	0.71 (0.17)	64.3 (18.9)
High	150 (16.9)	19 (12.7)	89 (59.3)	24 (16.0)	73 (48.7)	114 (76.0)	70 (46.7)	0.66 (0.20)	58.0 (20.1)
**Language**		*p* = 0.2028	*p* = 0.0016	*p* = 0.1652	*p* = 0.8768	*p* = 0.1540	*p* < 0.0001	*p* = 0.1592	*p* = 0.1218
German	548 (61.7)	81 (14.8)	264 (48.2)	70 (12.8)	214 (39.1)	409 (74.6)	201 (36.7)	0.71 (0.18)	64.0 (19.5)
French	340 (38.3)	40 (11.8)	127 (37.4)	33 (9.7)	131 (38.5)	268 (78.8)	171 (50.3)	0.69 (0.17)	61.9(18.9)

*1 missing

** 3 missing

VAS: visual analog scale

CC: chronic conditions

The observed mean HU and VAS values in all multimorbid patients were significantly lower than the values predicted by the general population and, moreover, were also significantly lower in multimorbid patients below 60 years old. We also found differences between observed and predicted values by sex. The difference between the present study’s observed and predicted mean values of HU and VAS were significant and are presented in Table 4.

**Table 4:** Observed and predicted mean health utility and VAS, and the differences between them in 888 multimorbid patients.

	**EQ-5D health utility**				**Visual analogue scale**			
	Observed (SD)	Predicted	Difference observed–predicted (SD)		Observed (SD)	Predicted	Difference observed–predicted (SD)	
**Total**	0.70 (0.18)	0.77	-0.07*** (0.18)		63.19 (19.25)	74.98	-11.8*** (20.3)	
**Sex**								
Women	0.67 (0.17)	0.77	-0.10*** (0.18)		60.55 (19.33)	74.85	-14.3***(20.6)	
Men	0.73 (0.17)	0.76	-0.03** (0.18)		66.03 (18.78)	75.11	-9.1*** (19.7)	
**Age group**								
< 60	0.63 (0.21)	0.82	-0.19*** (0.22)		55.67 (22.01)	82.93	-27.3*** (22.2)	
60–79	0.71 (0.17)	0.77	-0.06*** (0.18)		66.30 (18.73)	76.39	-11.0*** (18.9)	
≥ 80	0.71 (0.15)	0.73	0.02* (0.15)		62.91 (17.92)	68.87	-5.9*** (18.2)	
	**EQ-5D health utility**				**Visual analogue scale**			
	**Women**		**Men**		**Women**	**Men**		
**Age group**	Observed (SD)	Predicted	Observed (SD)	Predicted	Observed (SD)	Predicted	Observed (SD)	Predicted
< 60	0.62 (0.2)	0.82	0.64 (0.24)	0.82	55 (22.21)	83.2	56.8 (21.8)	82.5
60–79	0.67 (0.17)	0.77	0.74 (0.17)	0.88	62.6 (18.48)	76.3	67.6 (18.6)	76.4
≥ 80	0.68 (0.15)	0.73	0.75 (0.14)	0.88	60.6 (18.42)	68.6	66.0 (16.8)	69.2

*** p ≤ 0.0001

** p ≤ 0.001

* p ≤ 0.01

## Discussion

The present study showed that self-perceived HU and VAS values were significantly lower in multimorbid patients in primary care than in Switzerland’s general population, i.e., self-perceived values of HU and VAS in our sample were lower than predicted values, with a considerably greater difference in the case of VAS. Moreover, we found that in our sample, self-perceived HU and VAS values were considerably and significantly lower in multimorbid patients below 60 years old than in older ones, and in women than in men. Although several studies have assessed QoL in multimorbid patients, to the best of our knowledge, this study is the first to provide information about self-perceived HU and VAS values using the EQ-5D-3L instrument in multimorbid patients with at least three chronic conditions in a primary care setting.

The present study’s main results were that self-perceived HU and VAS values were significantly lower in multimorbid patients than in Switzerland’s general population [21], confirming our hypothesis. In fact, although we expected to see lower HU and VAS values in multimorbid patients than in the general population, the differences were surprisingly large, especially for the VAS score. This result could be explained by the fact that multimorbidity is associated with poor QoL [6] and that multiple factors relating to multimorbidity (i.e., disease burden, treatment burden) can affect daily activities and well-being, including loss of independence and autonomy. These factors can have a far greater impact on the QoL of multimorbid patients than they would on the general population, which faces them less frequently. Mittmann et al. showed that HU were lower in people with one chronic condition [28], and Manuel et al. showed that HU varied from one condition to another [23]. These studies suggested that multimorbidity may have a negative impact on perceived health outcomes and thus lead to a lower HRQoL resulting in lower HU and VAS. Indeed, multimorbid patients’ QoL may be affected more than that of people in the general population with just one chronic condition or without any. In other words, in many patients, multiple chronic diseases can interact with each other, compounding their effects rather than just existing as distinct disorders.

Another important result was that self-perceived HU and VAS values were considerably lower in multimorbid patients below 60 years old than older multimorbid patients; this trend was contrary to that observed in the general population, where HU and VAS become lower as participants get older [21]. Although the measure used was different, Manuel et al. showed that age had an independent effect on HU [23], such that as age increased, QoL became proportionately more affected. In our sample of multimorbid patients, self-perceived HU and VAS seemed to improve with age. This could be explained by the fact that as patients with coexisting multiple chronic conditions get older, they appear to become more accustomed to their diseases and to have adapted to them. Perhaps they simply accept them because they feel it is normal to have multiple chronic conditions as one ages and, therefore, they self-reported a better QoL. Another explanation could be that multimorbid people below 60 years old have specific types of chronic conditions, not necessarily just those acquired with age and, therefore, they have difficulty accepting their diseases. A last explanation could be that people with depression are more likely to perceive a worse QoL [29, 30]. Younger people with chronic conditions are indeed more likely to be depressed than older people [31], and this could explain why our study’s under-60 group mostly reported a worse QoL than the older participants.

With regards to the EQ-5D’s five different dimensions, the present study’s multimorbid patients reported “no problem” more frequently than both “moderate problem” and “severe problem” for mobility, self-care, usual activities, and anxiety/depression. However, they frequently reported a “moderate problem” with pain/discomfort. This result is concordant with a study of older adults in Spain by León-Salas et al. [32], and with the results obtained in the general Swiss population (although not for pain/discomfort) [21], suggesting that the trend remains the same despite the different populations. This result suggests that when we look at the five dimensions separately, our sample’s multimorbid patients felt little impact on their mobility, their ability to take care of themselves and carry out usual activities and their state of anxiety/depression, whereas they were more affected by pain/discomfort. This could be because multimorbid patients reported pain/discomfort more readily. As they perceived it, pain/discomfort was the domain which put the greatest limitations on their lives; it was the most important and least bearable for them. This result agrees with the study by Pinto-Meza et al. which showed that chronic pain played a major role in the loss of HRQoL [33]. In primary care, therefore, we should pay more attention to the QoL of multimorbid patients who are suffering from pain. On the other hand, most multimorbid patients in the present study did not report severe problems in the domains of mobility, self-care, usual activities, and anxiety/depression, which suggested that they were not necessarily the patients most affected by their chronic conditions. Moreover, the majority of our study population had a low severity index rating, suggesting that they were not as sick and impaired as we might have imagined. This could be explained by the fact that the study probably did not include many of the most severely impaired patients: enrollment in the study occurred at GPs’ practices and concerned all patients with at least three of a list of 75 chronic conditions. Indeed, our goal was not to only have the most impaired patients but to include all kinds of multimorbid patients with at least three chronic conditions. Many of the chronic conditions and risk factors observed were silent and had a low impact on their daily activities, autonomy, and independence. This result could suggest that most multimorbid patients who are able to get to their GPs’ practices are less affected by issues of mobility, self-care, being able to carry out usual activities, and anxiety/depression. They thus reported fewer of these as QoL problems. Had patients who could not attend their GPs’ practices been enrolled in the study (i.e. patients receiving regular home visits, living in nursing homes, or hospitalized), its results could have been significantly different.

Women reported significantly more problems than men in every health dimension but self-care. They also reported lower mean HU and VAS values than men, suggesting that multimorbid women’s overall HRQoL is more affected by their chronic conditions than multimorbid men’s. The study sample’s trend for HU by sex was the same as that for the general population [21]. This result is also in accordance with the studies by Wai Yang Loo et al. [34] and Scirè et al. [35], although their populations and HRQoL measurements were different. These two studies showed that female participants perceived a greater degree of physical impairment and poorer overall HRQoL than male participants. Their results can be explained by the fact that it has been consistently shown that women have poorer health than men [36–38], thus poorer health inevitably leads to poorer QoL. The fact that the women in the present study reported fewer problems in the dimension of self-care could be because they are better than men at taking care of themselves. Alternatively, they may not have reported problems of self-care in order to avoid having to ask for help in this domain.

The EQ-5D-3L instrument’s best possible, problem-free health status (i.e., “11111”) was reported by 13.6% in our sample of multimorbid patients. This could be because multimorbid patients suffering from chronic conditions that are not very symptomatic, and thus have little impact on their QoL, feel that they have no problems. Indeed, the present study’s criteria for patient inclusion defined multimorbidity as having at least three chronic conditions, but there were no criteria as to the severity or degree of impairment caused by each condition. However, we can only speculate on this, as the present study was unable to associate the different health states to clusters of patients with chronic conditions.

### Strengths and limitations

This national primary care study analyzed data from a representative sample of multimorbid patients with at least three chronic conditions, enrolled in GPs’ practices across Switzerland. Although several studies have previously assessed the QoL of multimorbid patients, to the best of our knowledge, this is the first to have assessed their self-perceived HU and VAS values using the EQ-5D-3L instrument in a primary care setting. Results showed considerable differences between the study population and the country’s general population.

However, this study has some limitations. First, the sample might not be representative of all multimorbid patients. GPs only recruited patients who came to their practices and who had at least three chronic conditions from a list of 75 provided to them. Therefore, the most impaired multimorbid patients, those with the biggest mobility problems (i.e., those cared for through home visits, in nursing homes, or hospitalized) were not included. These results should thus be interpreted with caution, bearing in mind the multimorbid patients who cannot attend GPs’ practices or who have rare chronic conditions.

Second, this study was unable to carry out any assessment of self-perceived HU by cluster of multimorbid patients with the same three (or more) chronic conditions, or by each condition’s degree of severity. Indeed, we were unable to define a unique multimorbid profile due to the high number of combinations of chronic conditions included in the study. However, the study did allow us to present an overall idea of multimorbid patients’ self-perceived HU.

Third, the general population sample against which we compared our data was from the French-speaking part of Switzerland and thus may not be entirely representative of that mainly German-speaking country.

## Conclusions

By using the EQ-5D-3L instrument’s questionnaire about health utilities and its visual analogue scale, in a primary care setting, our findings suggested that quality of life was considerably and significantly lower in multimorbid patients than in the general population. We believe that measuring self-reported quality of life is an important aid to understanding the impact of requests for care and, very probably the intensity of care provided to them and thus healthcare costs. Moreover, our results suggest that quality of life seemed to be significantly lower in a subgroup of patients below 60 years old and in women. For GPs’ daily practice, this may imply that multimorbid patients’ overall quality of life should be more carefully assessed, particularly in those two groups. Similarly, more attention should be given to the development of interventions that improve the quality of life of multimorbid patients, particularly when multimorbidity begins to affect them below 60 years old.

## Supporting information

S1 Table List of chronic conditions. (DOCX)

## Abbreviations:

EQ -5D-3L: The 3-level version of EQ-5D
QoL: Quality of Life
HRQoL: Health Related Quality of Life
HU: Health Utilities
VAS: Visual analogue scale
CIRCS: Cumulative illness rating scale
SI: Severity index
