# Supplementary material for: Comparing the self-perceived quality of life of multimorbid patients and the general population using the EQ-5D-3L
Source: PLoS One. 2017 Dec 19;12(12):e0188499. doi: 10.1371/journal.pone.0188499 (PMC5736180; doi:10.1371/journal.pone.0188499)
Supplement: S1 Table — (DOCX) [file pone.0188499.s001.docx]

# Code attribué au médecin de famille:

| **Code patient: Date de naissance:** |  | | |
| --- | --- | --- | --- |
| **General and unspecified** |  | **Respiratory** |  |
| Pain general/multiple sites | A01 □ | Chronic bronchitis | R79 □ |
| Malignancy not otherwise specified | A79 □ | Malignant neoplasm bronchus/lung | R84 □ |
| Secondary effect of trauma | A82 □ | Chronic obstructive pulmonary disease | R95 □ |
| Other: |  | Asthma | R96 □ |
| **Blood, Blood Forming Organs and Immune Mechanism** |  | Other: |  |
| HIV-infection/aids | B90 □ | **Eye** |  |
| Hodgkin's disease/lymphoma | B72 □ | Retinopathy | F83 □ |
| Malignant neoplasm blood other | B74 □ | Macular degeneration | F84 □ |
| Other: |  | Blindness | F94 □ |
| **Digestive** |  | Other: |  |
| Incontinence of bowel | D17 □ | **Ear** |  |

Malignant neoplasm stomach D74 □

Malignant neoplasm colon/rectum D75 □

Malignant neoplasm pancreas D76 □

Malignant neoplasms digest other/not otherwise specified D77 □ Irritable bowel syndrome D93 □

Chronic enteritis/ulcerative colitis D94 □ Other:

# Endocrine/Metabolic and Nutritional

Obesity T82 □

Diabetes insulin dependent T89 □

Diabetes non-insulin dependent T90 □

Gout T92 □

Malignant neoplasm thyroid T71 □

Other:

Hearing complaint H02 □

Tinnitus, ringing/buzzing ear H03 □

Deafness H86 □

Other:

# Cardiovascular

Risk factor cardiovascular disease K22 □

Ischaemic heart disease with angina K74 □

Ischaemic heart disease without angina K76 □

Atrial fibrillation/flutter K78 □

Pulmonary heart disease K82 □

Elevated blood pressure K85 □

Hypertension uncomplicated K86 □

Hypertension complicated K87 □

Cerebrovascular disease K91 □

Atherosclerosis/Peripheral vascular disease K92 □ Other:

# Neurological

Poliomyelitis N70 □

Malignant neoplasm nervous system N74 □

Multiple sclerosis N86 □

Parkinsonism N87 □

Epilepsy N88 □

Migraine N89 □

Trigeminal neuralgia N92 □

Abnormal involuntary movements N08 □

Peripheral neuritis/neuropathy N94 □

Pain face N03 □

Other:

# Skin

Chronic ulcer skin S97 □

Malignant neoplasm of skin S77 □

Other:

# Musculoskeletal

Malignant neoplasm musculoskeletal L71 □

Rheumatoid/seropositive arthritis L88 □

Osteoarthrosis of hip L89 □

Osteoarthrosis of knee L90 □

Osteoporosis L95 □

Other:

# Urological

Incontinence urine U04 □

Malignant neoplasm of bladder U76 □

Malignant neoplasm of kidney U75 □

Other:

# Psychological

Chronic alcohol abuse P15 □

Tobacco abuse P17 □

Drug abuse P19 □

Dementia P70 □

Organic psychosis other P71 □

Schizophrenia P72 □

Affective psychosis P73 □

Somatization disorder P75 □

Depressive disorder P76 □

Phobia/compulsive disorder P79 □

Personality disorder P80 □

Post-traumatic stress disorder P82 □

Mental retardation P85 □

Anorexia nervosa/bulimia P86 □

Psychosis not otherwise specified/other P98 □

Medication abuse P18 □

Memory disturbance P20 □

Other:

# Female Genital

Malignant neoplasm cervix X75 □

Malignant neoplasm breast female X76 □ Other:

# Male Genital

Malignant neoplasm prostate Y77 □

Other:
